# Comparative study of neuroimaging evaluation between idiopathic normal pressure hydrocephalus and progressive supranuclear palsy

**DOI:** 10.1002/alz.088065

**Published:** 2025-01-09

**Authors:** Shoya Inagawa, Ryo Yamamoto, Yuta Inagawa, Naoto Takenoshita, Shuhei Shibukawa, Daisuke Yoshimaru, Kazuhiro Saito, Soichiro Shimizu

**Affiliations:** ^1^ Tokyo Medical University, Tokyo Japan

## Abstract

**Background:**

Idiopathic normal pressure hydrocephalus (iNPH) and progressive supranuclear palsy (PSP) are considered to be different pathologies, but they have similar clinical features such as cognitive dysfunction and gait disturbance. Therefore, it is difficult to understand between the two diseases in actual clinical practice. The aim of study is whether the indivisual characteristic in MRI is useful to devide into two groups.

**Method:**

15 iNPH patients and 9 PSP patients were enrolled. The subjects were recruited retrospectively from those who had been diagnosed in our department. The MRI device was 3 T MAGNETOM Vida (SIEMENS), and the Evans index and DESH score were measured using T2WI/FLAIR. Furthermore, we used FreeSurfer for 3D‐T1WI to measure the volumes of the brainstem, corpus callosum, and ventricles. Each brain region was adjusted using the whole brain volume, multiple corrections were performed on the final remaining regions, and comparisons were made between the two groups.

**Result:**

There were no significant differences in brain stem volume between the two groups. However, Evans index (iNPH: 0.351 ± 0.0, PSP: 0.286 ± 0.0, p < 0.01), DESH score (iNPH: 6.87 ± 1.2, PSP: 2.44 ± 2.1), p<0.01), corpus callosum volume (iNPH:854.75±369.9 mm^3, PSP:532.16±186.1 mm^3, p<0.05), cerebrospinal fluid volume (iNPH:2220.26±657.5 mm^3, PSP :1544.38±338.1 mm^3, p<0.01), a significant difference was observed.

**Conclusion:**

MRI findings related to the ventricles may be differentiating both diseases.

